# Impact of gender differences on hemostasis in patients after coronary artery bypass grafts surgeries in the context of tranexamic acid administration

**DOI:** 10.1186/s13019-022-01874-y

**Published:** 2022-05-21

**Authors:** Enshi Wang, Yang Wang, Shengshou Hu, Su Yuan

**Affiliations:** 1 Department of Cardiovascular Surgery, Fuwai Hospital, Chinese Academy of Medical Sciences, Peking Union Medical College, Xicheng District, Beijing, 100037 China; 2 Medical Research and Biometrics Center, National Center for Cardiovascular Diseases, Xicheng District, Beijing, 100037 China; 3 Department of Anesthesiology, Fuwai Hospital, Chinese Academy of Medical Sciences, Peking Union Medical College, Xicheng District, Beijing, 100037 China

**Keywords:** Female, Blood transfusion, Coronary artery bypass grafting, Tranexamic acid, Propensity score matching

## Abstract

**Background:**

Sex differences present in the blood management of patients after coronary artery bypass grafts (CABG) surgeries. Tranexamic acid (TXA) performed well in maintaining hemostasis during and after surgeries. However, the impact of sex differences on blood control after CABG in patients who received TXA was not investigated.

**Methods:**

Overall, 29,536 patients undergoing CABG with TXA administration from 2009 to 2019 in our hospital were included. Propensity score matching was performed. Finally, 6808 males and 6808 females were matched based on 23 covariates.

**Results:**

Female patients had a 0.36-fold lower incidence of reoperations due to major hemorrhage or cardiac tamponade compared to males (1.3% vs. 2.0%, *p* = 0.001, OR = 0.64, 95%CI = 0.49–0.84). Females had a median of 100 ml less blood loss in 24 h (median 360 vs. 460 ml, *p* < 0.0001), 150 ml less in 48 h (median 580 vs. 730 ml, *p* < 0.0001), and 180 ml less in total (median 760 vs. 940 ml, *p* < 0.0001) than male patients. The red blood cell (RBC) transfusion rate in female was 1.53-fold higher than that in male (33.0% vs. 21.6%, OR = 1.53, 95% CI = 1.43–1.63, *p* < 0.0001). Females also had higher morbidities than males after CABGs.

**Conclusions:**

Females had less blood loss than males after CABG with the TXA treatment. Females still had a higher RBC transfusion rate after surgery. Morbidities in women were also higher than that in men.

**Supplementary Information:**

The online version contains supplementary material available at 10.1186/s13019-022-01874-y.

## Introduction

Gender differences are present in the outcomes of coronary artery bypass grafts (CABG) surgeries. Female patients undergoing CABG surgeries were likely to have higher mortality, more comorbidity than male patients [1–5]. The blood transfusion was common in CABG surgeries [6]. In some studies, the female patients had a lower risk of bleeding and reoperation after CABG [7, 8]. However, females were the independent risk factor for the red blood cell infusion in other studies after CABG [9, 10]. So the blood management was different between females and males in patients after CABG surgeries.

The tranexamic acid (TXA) was now used widely as an antifibrinolytic agent during cardiac surgery [11]. The TXA obtained obvious hemostasis in patients who underwent CABG surgeries with less blood loss and lower blood infusion rate [12]. However, the gender effects on blood management in patients receiving CABG surgeries in the context of TXA administration were not evaluated at present. This study aimed to investigate blood loss and transfusion differences between male and female patients with TXA administration during CABG surgeries.

## Materials and method

### Patients information

From January 1st, 2009 to December 31st, 2019, 30,259 patients undergoing CABG surgeries in the administration of TXA were included in this retrospective cohort study. Patients aged < 18 years old, enrolled in clinical trials, or with missing values were excluded from this study. Finally, a total of 29,536 (97.6%) patients were included in the following statistical analyses. Among them, there were 22,631 (76.6%) males and 6905 (23.4%) females. The females account for less than 1/4 total patients number (Table 1). The CABG surgeries in this study were not only the isolated CABGs but also CABGs with other surgeries. The high risks surgery included the previous cardiac surgery, emergent surgery, CABG combined with valve operation or CABG combined with aortic or arch operation. The open chamber surgeries included CABG combined with valve surgery or aortic surgery or aneurysm resection.

**Table 1:** Variables in the propensity score matching

Order	Variables
1	TXA(≥ 50 mg/kg, < 50 mg/kg)
2	Age
3	Body mass index
4	Left ventricular dysfunction
5	Diabetes by insulin
6	Hypertension
7	Chronic kidney disease
8	Peripheral vascular disease
9	Cerebrovascular accident
10	Previous cardiac surgery
11	Acute myocardial infarction
12	Aspirin within 5 days
13	Clopidogrel within 5 days
14	LMWH within 24 h
15	Hemoglobin (g/L)
16	Thrombocytopenia
17	International normalized ratio
18	Activated partial thromboplastin time
19	Operation year
20	High-risk operation
21	On-pump
22	Duration of surgery (min)
23	Heparin neutralization ratio

*TXA* tranexamic acid, *LMWH* low-molecular-weight heparin

The study was carried out according to the Declaration of Helsinki 1964 and its subsequent amendments. The Medical Ethics Committee of the Fuwai Hospital approved the protocol. The informed consent was waived because of the retrospective study.

### Blood control during operation

TXA was given after anesthesia induction at a median of 53.86 mg/kg with interquartile ranging from 41.09 mg/kg to 69.44 mg/kg. Cell salvage was used routinely for blood conservation (Fresenius Kabi C.A.T.S.®^*plus*^, Fresenius Kabi AG, Bad Homburg, Germany). The dose of heparin was given in 400 IU/kg to keep the activated clotting time (ACT) ranging from 400 to 420 s for on-pump CABGs, and 200 IU/kg for off-pump CABGs to keep ACT around 300 s. During the surgery process, additional heparins were given according to the dynamic change of ACT. The protamine was given at a ratio of 1:1 to heparin (1 mg protamine: 100 units of heparin) for neutralization. More protamine was added in consideration of bleeding, ACT and the recommendation of surgeons (Additional file 1).

### Outcome measurement

The primary outcome was red blood cell (RBC) exposure after CABG.

The secondary outcomes were fresh frozen plasma (FFP), platelet (PLT) infusion, the reoperation due to major hemorrhage or cardiac tamponade, blood loss in 24 h, 48 h and total blood loss after surgery.

The safety issues were hospital death, death during the initial 30 postoperative days, postoperative myocardial infarction, stroke, acute kidney injury (AKI), pulmonary embolism, seizure associated with TXA administration, hospital stay, and intensive care unit (ICU) stay time.

### Statistical analysis

The continuous variables in normal distribution were presented as means ± standard deviation (SD) for normal distribution and median and interquartile range (IQR) for non-normal distribution. Numbers and percentages were used for the categorical variables.

The demographic and perioperative information was compared between the male and female patients before propensity score matching (PSM) was performed. The student's t-test was chosen for normal-distribution continuous variables, Mann–Whitney U-test for non-normal distribution variables, and χ2 test or Fisher's exact test for categorical variables. After PSM, the paired-t test was performed for normal distribution data, the Wilcoxon rank test for non-normal distribution data, and the McNemar’s test for categorical data in the demographical and perioperative information. While the conditional logistic regression was performed in the binary outcome evaluation with an odds ratio (OR) and 95% confidence intervals (CI).

**Fig. 1:**
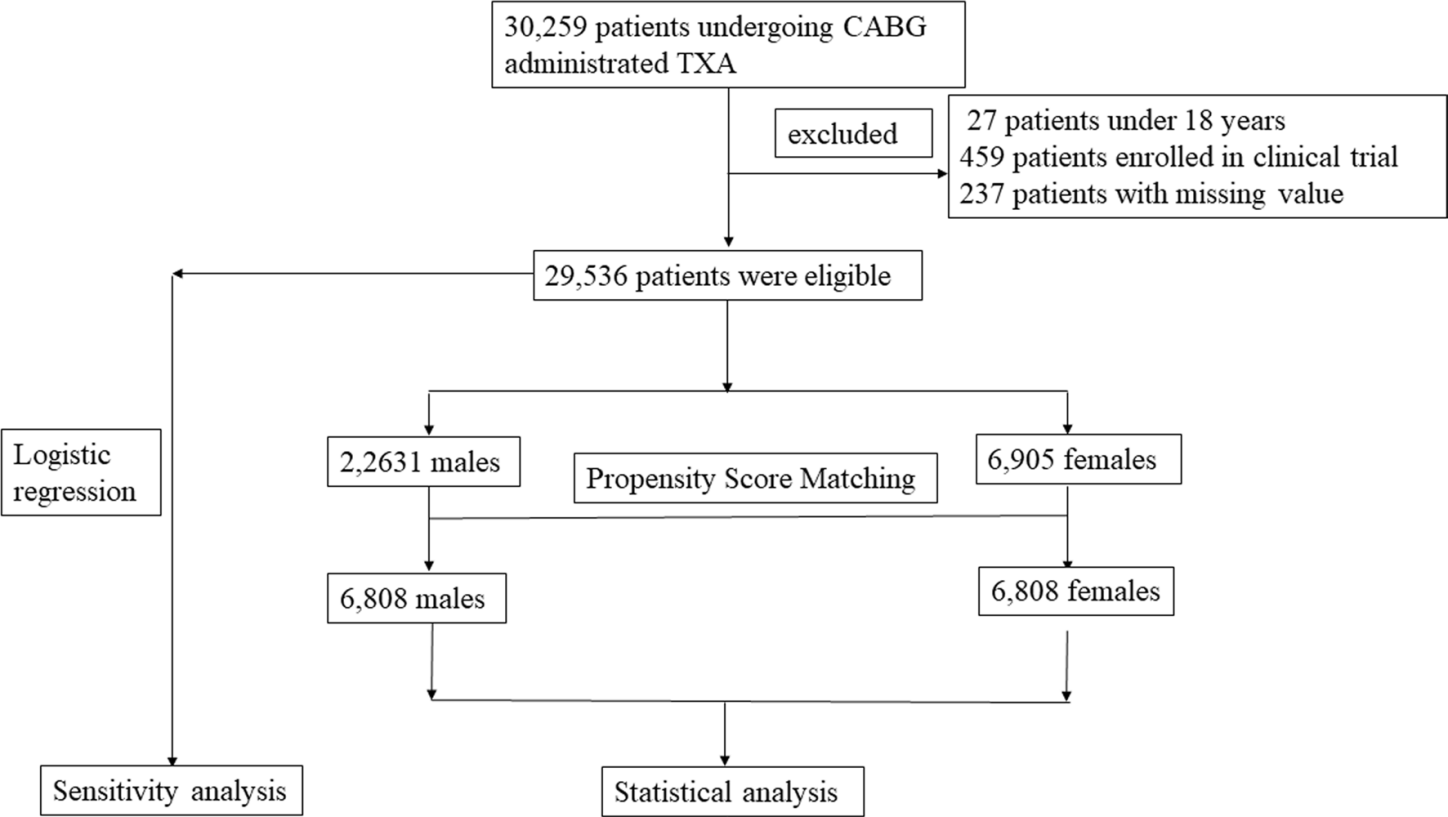
The flow sheet of different gender comparison in blood management of CABG patients with TXA administration. A total of 30,259 patients undergoing CABG surgeries in the administration of TXA were included in this retrospective cohort study. Patients aged < 18 years old, enrolled in clinical trials, or with missing values were excluded from this study. Finally, a total of 29,536 (97.6%) patients were included in the following PSM and sensitivity analyses. There were 22,631 (76.6%) males and 6905 (23.4%) females, respectively. Moreover, 6808 male and females patients were matched well. CABG, coronary artery bypass grafts; TXA, tranexamic acid; PSM, propensity score matching

**Fig. 2:**
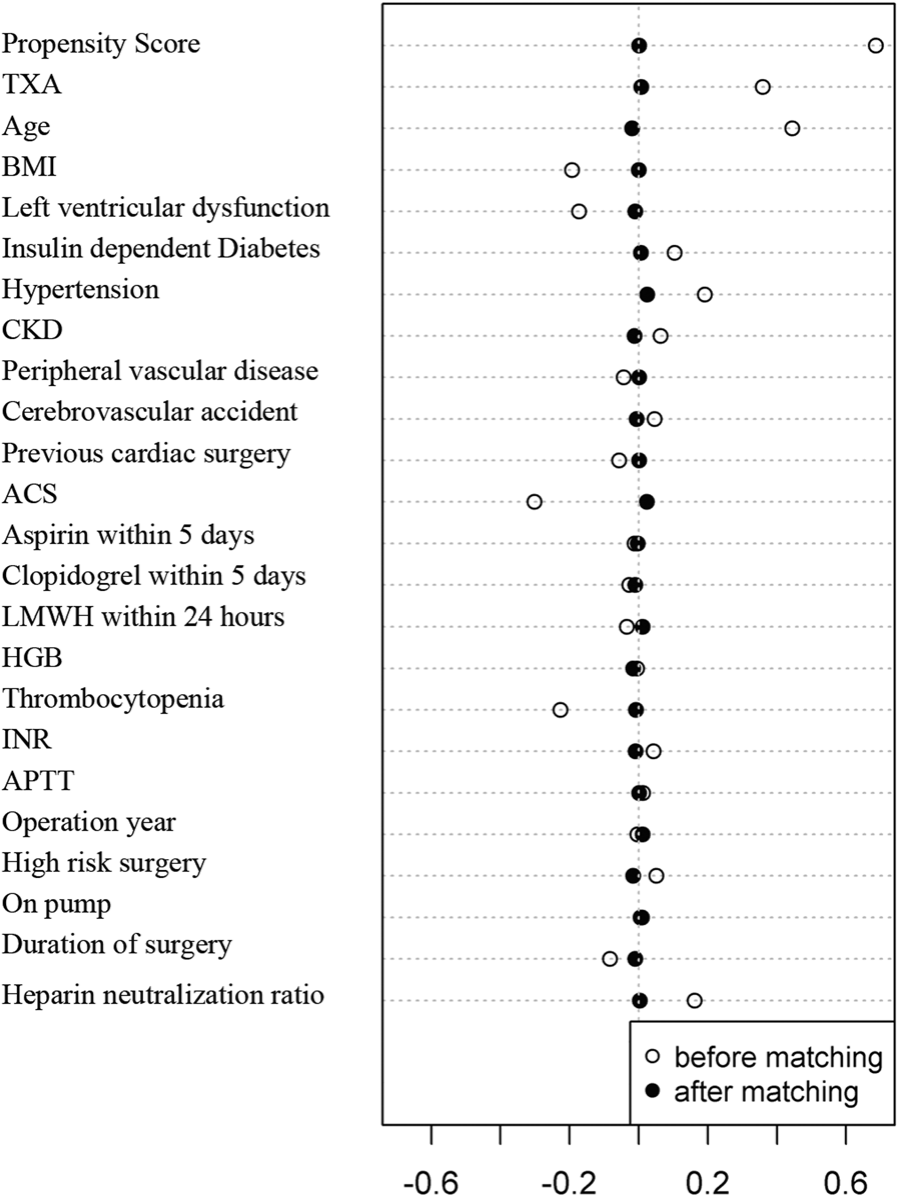
Propensity score matching results between male and female patients. The propensity score was matched well between male and female patients. The standardized difference was less than 0.1 in all 23 covariates

The propensity score matching was generated according to 1:1 matching between male and female patients in the entire CABG cohort in the context of TXA administration. The caliper width was 0.01, and the nearest-neighbor matching method without replacement was selected. A total of 23 variables was chosen for PSM based on the clinical and statistical significance (Table 1). In all, 6808 males and 6808 females were selected after being matched. Balances were well kept in the demographical and perioperative data with standardized differences < 0.1 (Table 2, 3, and Figs. 1, 2).

**Table 2:** Baseline information in male and female patients’ in tranexamic acid before and after propensity score matching

Characteristics	Before matching			After matching		
	Female (n = 6905)	Male (n = 22,631)	*p*-value	Male (n = 6808)	Female (n = 6808)	*p*-value
TXA (≥ 50 mg/kg), n (%)	4735(68.6)	11,753(51.9%)	< 0.0001	4613(67.8)	4638(68.1)	0.619
Age (y), mean ± SD	63.75 ± 7.54	60.39 ± 8.85	< 0.0001	63.71 ± 8.05	63.62 ± 7.49	0.406
BMI (kg/m^2^), mean ± SD	25.25 ± 3.30	25.88 ± 3.11	< 0.0001	25.27 ± 3.03	25.28 ± 3.29	0.910
BSA(m^2^), mean ± SD	1.62 ± 0.13	1.85 ± 0.15	< 0.0001	1.69 ± 0.11	1.69 ± 0.12	0.760
LV dysfunction (ejection fraction < 40%), n (%)	157(2.3)	1099(4.9)	< 0.0001	161(2.4)	157(2.3)	0.862
Preexisting medical conditions, n (%)						
Insulin dependent Diabetes	959(13.9)	2331(10.3)	< 0.0001	928(13.6)	923(13.6)	0.920
Hyperlipidemia	4673(67.7)	15,330(67.7)	< 0.0001	4583(67.3)	4610(67.7)	0.636
Hypertension	4796(69.5)	13,729(60.7)	< 0.0001	4702(69.1)	4705(69.1)	0.969
Chronic kidney disease	581(8.4)	1507(6.7)	< 0.0001	554(8.1)	557(8.2)	0.950
Peripheral vascular disease	630(9.1)	2348(10.4)	0.003	653(9.6)	626(9.2)	0.442
Cerebrovascular accident	989(14.3)	2875(12.7)	< 0.0001	986(14.5)	974(14.3)	0.789
Previous cardiac surgery	189(2.7)	830(3.7)	< 0.0001	195(2.9)	188(2.8)	0.755
Acute coronary syndrome	917(13.3)	5323(23.5)	< 0.0001	864(12.7)	915(13.4)	0.173
Preoperative IABP	71(1.0)	300(1.3)	0.052	71(1.0)	68(1.0)	0.865
Time between CAG and operation less than 3 days	254(3.7)	825(3.6)	0.898	245(3.6)	251(3.7)	0.819
Preoperative medications, n (%)						
Aspirin within last 5 days	740(10.7)	2508(11.1)	0.396	719(10.6)	738(10.8)	0.617
Clopidogrel within last 5 days	867(12.6)	3050(13.5)	0.048	849(12.5)	866(12.7)	0.677
Ticagrelor within last 5 days	48(0.7)	153(0.7)	0.866	47(0.7)	48(0.7)	1.000
LWMH within 24 h	1613(23.4)	5615(24.8)	0.014	1576(23.1)	1591(23.4)	0.776
Preoperative laboratory tests						
HGB (g/L), mean ± SD	134.54 ± 13.98	134.60 ± 14.15	0.737	134.55 ± 14.15	134.53 ± 13.97	0.945
PLT(< 150 × 10^–9^/L), n (%)	492(7.1)	2931(13.0)	< 0.0001	497(7.3)	492(7.2)	0.891
INR(> 1.20), n (%)	121(1.8)	270(1.2)	< 0.0001	119(1.7)	118(1.7)	1.000
APTT(> 43.5 s), n (%)	425(6.2)	1327(5.9)	0.370	421(6.2)	418(6.1)	0.944
Propensity Score, mean ± SD	0.30 ± 0.13	0.30 ± 0.13	0.004	0.30 ± 0.12	0.30 ± 0.12	< 0.0001

*TXA* tranexamic acid, *BMI* body mass index, *BSA* body surface area, *LV* left ventricle, *IABP* intra-aortic balloon pump, *CAG* coronary angiography, *LMWH* low-molecular-weight heparin, *HGB* hemoglobin, *PLT* platelet, *INR* international normalized ratio, *APTT* activated partial thromboplastin time, *SD* standard deviation

**Table 3:** Operation characteristics between the male and female patients in tranexamic acid before and after propensity score matching

Surgery data	Before matching			After matching		
	Female (n = 6905)	Male (n = 22,631)	*p*-value	Female (n = 6808)	Male (n = 6808)	*p*-value
CABGs by experienced surgeons (≥ 100 CABGs / year), n (%)	3692(53.5)	12,186(53.8)	0.581	3628(53.3)	3641(53.5)	0.838
Operation year (2009–2014), n (%)	2561(37.1)	8437(37.3)	0.773	2509(36.9)	2452(36.0)	0.319
Operation year (2015–2019), n (%)	4344(62.9)	14,194(62.7)	0.773	4299(63.1)	4356(64.0)	0.319
High risk operation, n (%)	1420(20.6)	4190(18.5)	< 0.0001	1397(20.5)	1408(20.7)	0.832
Emergent surgery, n (%)	233(3.4)	647(2.9)	0.027	226(3.3)	212(3.1)	0.529
Elective, n (%)	6672(96.6)	21,984(97.1)	0.027	6582(96.7)	6596(96.9)	0.529
Isolated CABG, n (%)	5710(82.7)	19,110(84.4)	0.001	5632(82.7)	5612(82.4)	0.667
On-pump, n (%)	4365(63.2)	14,202(62.8)	0.488	4312(63.3)	4328(63.6)	0.790
Open-chamber, n (%)	1172(17.0)	3411(15.1)	< 0.0001	1153(16.9)	1172(17.2)	0.681
Heparin neutralization ratio, mean ± SD	1.41 ± 0.50	1.33 ± 0.46	< 0.0001	1.40 ± 0.50	1.40 ± 0.48	0.766
Duration of surgery (min), mean ± SD	286.36 ± 84.72	293.40 ± 85.88	< 0.0001	286.48 ± 84.65	290.43 ± 87.38	0.007

High risk: previous cardiac surgery, emergent surgery, CABG combined with valve operation and CABG combined with aortic or arch operation; Open chamber: CABG combined with valve surgery or aortic surgery or aneurysm resection; SD, standardized deviation. High risks operation: previous cardiac surgery, emergent surgery, CABG combined with valve operation or CABG combined with aortic or arch operation

*CABG* coronary artery bypass graft

For sensitivity analysis, the binary logistic regression was performed in the entire cohort receiving TXA during CABG surgeries. The gender plus 23 variables mentioned above were chosen as covariates. The binary outcomes were taken as dependent variables. The "enter" method was used, and adjusted OR and 95% CI of outcome variables were generated.

*p* < 0.05 was considered statistically significant. All statistical analyses were carried out with IBM SPSS Statistics for Windows, version 22.0 (IBM Corp., Armonk, NY, USA).

## Results

### Baseline comparison before PSM

More females received a high dose of TXA (68.6% vs. 51.9% in TXA ≥ 50 mg/kg) than males (Table 2). Also, female patients were older (63.75 ± 7.54 vs. 60.39 ± 8.85, *p* < 0.0001) and had lower BMI (25.25 ± 3.30 vs. 25.88 ± 3.11, *p* < 0.0001) than male patients at the age of CABG surgeries (Table 2). The BSA in male was 1.85 ± 0.15 m^2^ which was larger than 1.62 ± 0.13 m^2^ in females (*p* < 0.0001). Also, females undergoing CABG in this study had a higher incidence of insulin-dependent diabetes, hypertension, CKD, and CVA (*p* < 0.0001) (Table 2). The females had no difference in HGB level (134.54 ± 13.98 vs. 134.60 ± 14.15, *p* = 0.737) and less thrombocytopenia (7.1% vs. 13.0%, *p* < 0.0001) compared to males before operation (Table 2). More high-risk surgeries were performed in females than males administrated with TXA(20.6% vs. 18.5%, *p* < 0.0001) (Table 3).

### Primary and secondary outcome evaluation: postoperative blood transfusion and blood loss

#### Results after PSM

In this study, the RBC transfusion rate in females was 1.53-fold higher than that in males (33.0% vs. 21.6%, OR = 1.53, 95% CI = 1.43–1.63, *p* < 0.0001) (Table 4). The FFP infusion rate in females was 0.37-fold lower than that in males (10.1% vs. 16.0%, OR = 0.63, 95% CI = 0.58–0.70, *p* < 0.0001) (Table 4). As for the PLT transfusion rate, there was no differences between the male and female patients (2.7% vs. 2.3%, OR = 1.17, 95% CI = 0.94–1.45, *p* < 0.0001) (Table 4).

**Table 4:** Primary and secondary outcomes in male and female patients in tranexamic acid after PSM

Outcomes	After PSM				After Logistic regression	
	Female (n = 6808)	Male (n = 6808)	OR (95%CI)	*p* value	β (95% CI)	*p* value
Primary outcome						
RBC, n (%)	2246(33.0)	1469(21.6)	1.53 (1.43–1.63)	< 0.0001	2.12 (1.98–2.27)	< 0.0001
Secondary outcome						
FFP, n (%)	689(10.1)	1088(16.0)	0.63(0.58–0.70)	< 0.0001	0.60(0.55–0.66)	< 0.0001
PLT, n (%)	181(2.7)	155(2.3)	1.17(0.94–1.45)	0.156	1.35(1.11–1.64)	0.003
Reoperation due to major hemorrhage or cardiac tamponade, n (%)	88(1.3)	137(2.0)	0.64(0.49–0.84)	0.001	0.67(0.53–0.85)	0.001
Blood loss in 24 h after surgery (mL), median (IQR)	360(260–490)	460(330–610)		< 0.0001	− 106.61(− 114.33 ~ − 98.89)	< 0.0001
Blood loss in 48 h after surgery (ml), median (IQR)	580(440–760)	730(550–940)		< 0.0001	− 156.18(− 166.69 ~ − 145.67)	< 0.0001
Total Blood loss after surgery(ml), median (IQR)	760(570–1070)	940(700–1310)		< 0.0001	− 206.33(− 228.66 ~ − 184.00)	< 0.0001

*TXA* tranexamic acid, *RBC* red blood cell, *FFP* fresh frozen plasma, *PLT* platelet, *OR* odds ratio, *CI* confidence interval, *IQR* Interquartile range, *SD* standard deviation

Generally, female patients had less blood loss than males after CABG surgeries with the administration of TXA. Female patients had a 0.36-fold lower incidence of reoperations due to major hemorrhage or cardiac tamponade compared to males (1.3% vs. 2.0%, *p* = 0.001, OR = 0.64, 95%CI = 0.49–0.84) (Table 4). Also, females had a median of 100 ml less blood loss in 24 h [median 360 ml, IQR (260–490 ml) vs. median 460 ml, IQR (330–610 ml), *p* < 0.0001], 150 ml less in 48 h [median 580 ml, IQR (440–760 ml) vs. median 730 ml, IQR (550–940 ml), *p* < 0.0001], and 180 ml less in total [median 760 ml, IQR (570–1070 ml) vs. median 940 ml, IQR (700–1310 ml), *p* < 0.0001] than male patients (Table 4).

#### Similar results were found in the sensitivity analyses

### Safety issues: post-CABG events between male and female patients

#### Results after PSM

There were no differences in the ICU and the hospital stay time between males and females. However, the females had higher risks of hospital death (0.4% vs. 0.2%, OR = 1.93, 95% CI = 1.04–3.61, *p* < 0.0001) and death during the initial 30 postoperative days (0.9% vs. 0.6%, OR = 1.49, 95% CI = 1.01–2.19, *p* < 0.0001) (Table 5). Also, the risks of stroke and AKI rise 1.76-fold (*p* < 0.0001) and 1.24-fold (*p* = 0.005) in the female (Table 5), respectively. No difference was observed in seizure between male and female patients (0.2% vs. 0.2%, *p* = 0.706).

**Table 5:** Safety issues in male and female patients in tranexamic acid after PSM

Postoperative course	After PSM				After Logistic regression	
	Female (n = 6808)	Male (n = 6808)	OR (95%CI)	*p* value	β (95% CI)	*p* value
Intensive care (h), median (IQR)	48(24–96)	48(24–96)		0.705	− 0.201(− 2.419 ~ 2.018)	0.859
Hospital stay (d), mean ± SD	16.93 ± 7.34	17.18 ± 9.61		0.557	− 0.130(− 0.361 ~ 1.000)	0.267
Adverse events after surgery, n (%)						
Hospital death	29(0.4)	15(0.2)	1.93(1.04–3.61)	0.038	1.91(1.16–3.14)	0.011
Myocardial infarction	462(6.8)	474(7.0)	0.98(0.86–1.11)	0.695	1.02(0.91–1.27)	0.746
Stroke	109(1.6)	62(0.9)	1.76(1.29–2.40)	< 0.0001	1.59(1.25–2.03)	< 0.0001
Acute kidney injury	389(5.7)	315(4.6)	1.24(1.06–1.43)	0.005	1.31(1.15–1.50)	< 0.0001
Pulmonary embolism	8(0.1)	11(0.2)	1.38(0.55–3.42)	0.493	1.62(0.76–3.46)	0.212
Death from any cause within 30 days	64(0.9)	43(0.6)	1.49(1.01–2.19)	0.044	1.58(1.14–2.18)	0.006
Seizure	13(0.2)	15(0.2)	0.87(0.41–1.82)	0.706	1.26(0.65–2.44)	0.499

*TXA* tranexamic acid, *RBC* red blood cell, *FFP* fresh frozen plasma, *PLT* platelet, *OR* odds ratio, *CI* confidence interval, *IQR* interquartile range, *SD* Standard deviation

#### Similar results were found in the sensitivity analyses

## Discussion

The gender differences in blood management after CABG surgery were reported before [6, 8, 10, 13]. The TXA as an antifibrinolytic agent was widely used in CABGs to maintain hemostasis during the perioperative period and proved to be effective [11, 12]. However, the sex differences in blood control with TXA management in CABG surgeries was still not investigated. Our study focused on patients who received TXA administrations during CABG and further explored the sex differences in blood management and postoperative outcomes in CABG surgeries. In this study, the RBC transfusion rate in females with the management of TXA in CABG surgery was higher than that in males. However, there was lower FFP exposure in females corresponding to the bleeding volume. Females had less blood loss and re-exploration rate than males after surgery. Also, females had a higher risk of hospital death, death during the initial 30 postoperative days, stroke, and AKI than males.

Most studies reported that females underwent CABG at an older age and presented more comorbidities [1, 14–19]. In an Australian retrospective cohort study, 21,534 patients undergoing CABGs were included [1]. Among them, 22.2% were female. Among them, 22.2% were female. Female patients were generally older (mean age, 68 vs. 65 years, *p* < 0.001) and presented more often with hypertension, diabetes mellitus, and cerebrovascular disease [1]. In this study, females accounted for 23.4% of the patients' number. Females were with older age, smaller BMI, and higher incidences of insulin-dependent diabetes, hypertension, chronic kidney disease, and cerebrovascular disease. This was in accordance with literature published before [1, 3, 20].

Some researchers discovered that the reoperation rate for bleeding after CABG was lower in women than in men [1, 4, 21, 22]. In retrospective research by Saxena et al. [1], females had a lower reoperation rate than males (1.9% vs. 2.5%) and reduced the risk of reoperation by 0.57-fold. Alam et al. found that females also had a lower incidence of reoperation for bleeding (4.2% vs. 4.5%, OR = 0.78) [4]. Mehta et al. revealed that women had a lower risk to bleed after CABG after analyzing 528,686 CABG patients in the Society of Thoracic Surgeons National Cardiac Database from 2004 to 2007 [22]. In our study, the administration of TXA did not influence the gender difference in the bleeding risk after CABGs. The females decreased a 0.36-fold risk of reoperation for cardiac tamponade or major bleeding compared to males. Swaminathan et al. [7] included 2,272,998 patients who underwent isolated CABG surgery, 27.4% of whom were female. The bleeding after surgery was decreased 0.17-fold by females [7]. Our study revealed similar results that the female chest tube drainage after CABG decreased significantly.

In this study, the females had a higher risk of RBC transfusion despite less blood loss. Many studies reported that female was an independent risk factor for RBC transfusion after CABG [9, 10, 15, 23–25]. Schwann and coworkers [5] found that patients with a low BMI were more hemodiluted and received more transfusions during and after the operation. In this study, the BMI in females was smaller than in males (mean 25.25 vs. 25.88, *p* < 0.0001). Also, factors such as cardiopulmonary bypass (CPB) or crystalloid solution infusion could influence the hemodilution extent differently by gender. Scott et al. [9] found that hemodilution during CPB was more pronounced in women than in men (lowest hematocrit on CPB, 23.7% versus 27.6%). In our hospital, the postoperative transfusion threshold was 80 g/L in hemoglobin and was not defined differently according to sex. So based on the more dilution during surgery, women undergoing surgery have a greater likelihood of receiving a blood transfusion than men after surgery [9]. It is likely that at equivalent hematocrits and weights, the total red cell volume for a woman is less than that of a man because of lower lean mass and plasma volume. So any dilutional anemia incurred by undergoing CABG would have a more profound effect on a woman and make them more likely to undergo transfusion [13]. So the female gender was an independent risk factor for RBC transfusion that was not mainly determined by the postoperative blood loss volume.

Koch et al. [25] revealed no gender differences were found in the transfusion of PLT and cryoprecipitate. FFP transfusions are commonly used in our hospital to control bleeding after surgery, and surgical re-exploration due to major bleeding or cardiac tamponade was always accompanied by FFP infusion. Therefore, it is not surprising that these blood products are a marker of postoperative bleeding. So the females received a lower incidence of FFP transfusion corresponding to less blood loss after CABGs in this study.

Only a few articles published the results of gender differences in bleeding and blood transfusion after CABGs concomitantly [1, 25]. Koch et al. [25] reported no gender differences in reoperation for tamponade or bleeding combined with lower RBC transfusion in women. Saxena and her colleges found that the females had a 0.43-fold risk of reoperation for bleeding but a 1.77-fold increased risk for RBC transfusion after CABGs in a cohort of 21,534 patients [1]. Nevertheless, the bleeding after surgery in these studies was just qualitative but not quantified. In the study here, we analyzed both bleeding and blood transfusion simultaneously and showed the accurate volume of chest tube drainage in different gender. It would give us a comprehensive and refined perspective on blood management in CABG patients.

Females could increase the risk of hospital death or death during the initial 30 postoperative days in this study. The results were in accordance with the literature before [2–5, 7, 14, 18, 19, 25]. Shi et al. [3] reported a meta-analysis incorporating 5,008,262 patients after CABG. The overall 30-day mortality was 4.9% in women versus 3.3% in men. In another meta-analysis by Robinson et al. [5], 903,346 patients after CABG surgeries were included for analysis. Females were at a 1.77-fold higher risk for operative mortality and a 1.16-higher risk of late mortality compared to males. In our hospital cohort, either the hospital mortality or death during the initial 30 postoperative days was lower than that published before. It was also well-known that the morbidity after CABG was also higher in females than in males [5]. In the meta-analysis by Robinson and his colleges, stroke risk was also increased by 1.35-fold in females. Swaminathan et al. [7] collected 2,272,998 CABG patients and found that the risk of AKI after surgery was increased in the female gender by a 1.08-fold. In the postoperative event in this cohort, we also found that stroke and AKI were higher after surgery in females than in males.

The TXA was widely used in cardiac surgery as a substitute for aprotinin after 2008 [26]. Series studies validated its specialty in reasonable blood control and low comorbidity [11, 12]. However, the thromboembolic events in the TXA were still considered by anesthesiologists and surgeons. Literature before mainly focused on the impact of gender differences on bleeding or blood transfusion in CABG patients, in which the effect of TXA was unclear. This study focused on patients who received TXA administration during CABGs and investigated the sex impact on hemostasis in this context. The results here were not different from previous literature, which meant that the use of TXA would not influence the gender difference in the CABG outcomes.

There were several limitations in this study. First, this was a retrospective study that included 29,536 patients. The patients' baseline information was not intact as the prospective clinical trial; Second, all patients received TXA in this study, so we could not determine whether there was a sex-treatment interaction; Finally, the PSM were used to simulate a RCT. However, the confounding factors were still present possibly.

## Conclusion

In this study, females had less blood loss after CABG and thereby a lower FFP transfusion rate. However, it could not change that females were the risk factor for RBC transfusion after surgery. Meanwhile, morbidities in women were also higher than that in men.

## Supplementary Information

**Additional file 1:** Supplementary Information.

## Data Availability

The datasets used and/or analysed during the current study are available from the corresponding author on reasonable request.

## Abbreviations

CABG: Coronary artery bypass grafts
TXA: Tranexamic acid
ACT: Activated clotting time
RBC: Red blood cell
FFP: Fresh frozen plasma
PLT: Platelet
AKI: Acute kidney injury
ICU: Intensive care unit
SD: Standard Deviation
IQR: Interquartile range
PSM: Propensity score matching
OR: Odds ratio
CI: Confidence interval
BMI: Body mass index
CKD: Chronic kidney disease
CVA: Cerebrovascular accident
HGB: Hemoglobin
INR: International normalized ratio
APTT: Activated partial thromboplastin time
HNR: Heparin neutralization ratio

## References

[1] Saxena A, Dinh D, Smith JA, Shardey G, Reid CM, Newcomb AE (2012). Sex differences in outcomes following isolated coronary artery bypass graft surgery in Australian patients: analysis of the Australasian Society of Cardiac and Thoracic Surgeons cardiac surgery database. Eur J Cardiothorac Surg..

[2] Huckaby LV, Seese LM, Sultan I, Gleason TG, Wang Y, Thoma F (2020). The impact of sex on outcomes after revascularization for multivessel coronary disease. Ann Thorac Surg..

[3] Shi D, Zhang B, Motamed M, Lee S, Wang P, McLaren C (2020). Higher mortality in women after coronary artery bypass: meta-analysis and bias analysis of confounding. Ann Thorac Surg..

[4] Alam M, Lee VV, Elayda MA, Shahzad SA, Yang EY, Nambi V (2013). Association of gender with morbidity and mortality after isolated coronary artery bypass grafting. A propensity score matched analysis. Int J Cardiol..

[5] Bryce RN, Naik A, Rahouma M, Morsi M, Wright D, Hameed I (2021). Sex differences in outcomes following coronary artery bypass grafting: a meta-analysis. Interact Cardiovasc Thorac Surg..

[6] Murphy GJ, Reeves BC, Rogers CA, Rizvi SI, Culliford L, Angelini GD (2007). Increased mortality, postoperative morbidity, and cost after red blood cell transfusion in patients having cardiac surgery. Circulation.

[7] Swaminathan RV, Feldman DN, Pashun RA, Patil RK, Shah T, Geleris JD (2016). Gender differences in in-hospital outcomes after coronary artery bypass grafting. Am J Cardiol..

[8] Ter Woorst JF, van Straten AHM, Houterman S, Soliman-Hamad MA (2019). Sex difference in coronary artery bypass grafting: preoperative profile and early outcome. J Cardiothorac Vasc Anesth.

[9] Scott BH, Seifert FC, Glass PSA, Grimson R (2003). Blood use in patients undergoing coronary artery bypass surgery: impact of cardiopulmonary bypass pump, hematocrit, gender, age, and body weight. Anesth Analg.

[10] Rogers MA, Blumberg N, Saint SK, Kim C, Nallamothu BK, Langa KM (2006). Allogeneic blood transfusions explain increased mortality in women after coronary artery bypass graft surgery. Am Heart J.

[11] Henry DA, Carless PA, Moxey AJ, O'Connell D, Stokes BJ, Fergusson DA (2021). Anti-fibrinolytic use for minimising perioperative allogeneic blood transfusion. Cochrane Database Syst Rev..

[12] Myles PS, Smith JA, Painter T (2017). Tranexamic acid in patients undergoing coronary-artery surgery. N Engl J Med.

[13] Williams ML, Trivedi JR, Doughtie C, Slaughter MS (2011). Is female sex an independent risk factor for perioperative transfusion in coronary artery bypass graft surgery?. J Am Coll Surg.

[14] Woods SE, Noble G, Smith JM, Hasselfeld K (2003). The influence of gender in patients undergoing coronary artery bypass graft surgery: an eight-year prospective hospitalized cohort study. J Am Coll Surg.

[15] Ranucci M, Pazzaglia A, Bianchini C, Bozzetti G, Isgro G (2008). Body size, gender, and transfusions as determinants of outcome after coronary operations. Ann Thorac Surg.

[16] Schwann TA, Habib RH, Zacharias A, Parenteau GL, Riordan CJ, Durham SJ (2001). Effects of body size on operative, intermediate, and long-term outcomes after coronary artery bypass operation. Ann Thorac Surg..

[17] Reeves BC, Ascione R, Chamberlain MH, Angelini GD (2003). Effect of body mass index on early outcomes in patients undergoing coronary artery bypass surgery. J Am Coll Cardiol.

[18] Toumpoulis IK, Anagnostopoulos CE, Balaram SK, Rokkas CK, Swistel DG, Ashton RC (2006). Assessment of independent predictors for long-term mortality between women and men after coronary artery bypass grafting: are women different from men?. J Thorac Cardiovasc Surg.

[19] Vaccarino V, Abramson JL, Veledar E, Weintraub WS (2002). Sex differences in hospital mortality after coronary artery bypass surgery: evidence for a higher mortality in younger women. Circulation.

[20] D'Agostino RB, Vasan RS, Pencina MJ, Wolf PA, Cobain M, Massaro JM (2008). General cardiovascular risk profile for use in primary care: the Framingham Heart Study. Circulation.

[21] Parolari A, Dainese L, Naliato M, Polvani G, Loardi C, Trezzi M (2008). Do women currently receive the same standard of care in coronary artery bypass graft procedures as men?. A propensity analysis Ann Thorac Surg.

[22] Mehta RH, Sheng S, O'Brien SM, Grover FL, Gammie JS, Ferguson TB (2009). Reoperation for bleeding in patients undergoing coronary artery bypass surgery: incidence, risk factors, time trends, and outcomes. Circ Cardiovasc Qual Outcomes.

[23] Shevde K, Pagala M, Kashikar A, Tyagaraj C, Shahbaz N, Iqbal M (2000). Gender is an essential determinant of blood transfusion in patients undergoing coronary artery bypass graft procedure. J Clin Anesth.

[24] Scott BH, Seifert FC, Glass PS (2003). Does gender influence resource utilization in patients undergoing off-pump coronary artery bypass surgery?. J Cardiothorac Vasc Anesth.

[25] Koch CG, Khandwala F, Nussmeier N, Blackstone EH (2003). Gender and outcomes after coronary artery bypass grafting: a propensity-matched comparison. J Thorac Cardiovasc Surg.

[26] Schneeweiss S, Seeger JD, Landon J, Walker AM (2008). Aprotinin during coronary-artery bypass grafting and risk of death. N Engl J Med.

