# Supplementary material for: Impact of gender differences on hemostasis in patients after coronary artery bypass grafts surgeries in the context of tranexamic acid administration
Source: J Cardiothorac Surg. 2022 May 21;17:123. doi: 10.1186/s13019-022-01874-y (PMC9123662; doi:10.1186/s13019-022-01874-y)
Supplement: Supplementary file 1 — Additional file 1: Supplementary Information. [file 13019_2022_1874_MOESM1_ESM.docx]

Supplementary Material

1. Definition of variables
   1. Chronic kidney disease (CKD): Kidney function is defined as normal with a GFR of more than 89 mL/min/1.73 m^2^ (stage I), minimally reduced with a GFR between 60 and 89 mL/min/1.73 m^2^ (stage II), moderately reduced with a GFR between 30 and 59 mL/min/1.73 m^2^ (stage III), severely reduced with a GFR between 15 and 29 mL/min/1.73 m^2^ (stage IV), and end-stage kidney failure with a GFR below 15 mL/min/ 1.73 m^2^ or renal replacement therapy (stage V). In this study, CKD was defined as kidney function≥ stage III ^2^.
   2. Open chamber operation: CABG with valve operation, CABG with ascending aortic or aortic arch operation and CABG with aneurysm resection were included in open chamber operation^4^.
2. Primary endpoint: RBC transfusion after CABG surgeries. In our hospital, the packed red blood cell was transfused if the hemoglobin concentration below 7 g/dl during the cardiopulmonary bypass period and below 8 g/dl after operation^9^.
3. Secondary endpoint:
   1. FFP and PLT transfusion after CABG surgeries. The decision to transfuse fresh frozen plasma, platelets or surgical re-exploration was made under the consideration of blood loss, thromboelastogram ((TEG, Haemoscope Corporation, USA)) results, and patients’ hemodynamic stability by recovering unit physicians and surgeons together.
   2. Reoperation due to major hemorrhage or cardiac tamponade.
   3. Blood loss in 24 hours, 48 hours and total blood loss after surgery.
4. Safety issues:
   1. Hospital death: all-cause mortality after surgery during hospitalization.
   2. Perioperative myocardial infarction: is diagnosed by an Isolated elevation of CK-MB to ≥10×99th percentile upper reference limit (URL) or cTn (I or T) to ≥70×URL during the first 48 h following CABG surgery with or without ECG or imaging changes of MI^5,6^
   3. Stroke: A new-onset of neurological deficit symptoms secondary to cerebral infarction or hemorrhage on CT scan, and persisted for more than 24 h ^7^. A neurologist confirmed the diagnosis by reviewing the hospital records in all cases.
   4. Acute kidney injury(AKI): According to RIFLE (Risk, Injury, Failure, Loss, and End-stage kidney disease) criteria^8^, the definition of AKI was that the increased serum creatine×2 or GFR decrease > 50%.
   5. Pulmonary embolism: high probability VQ scan or documented on pulmonary angiogram.
5. References

1. Levey AS, Bosch JP, Lewis JB, Greene T, Rogers N, Roth D. A more accurate method to estimate glomerular filtration rate from serum creatinine: a new prediction equation. Modification of Diet in Renal Disease Study Group. *Ann Intern Med.* 1999;130(6):461-470.

2. Fox CS, Muntner P, Chen AY, Alexander KP, Roe MT, Wiviott SD. Short-term outcomes of acute myocardial infarction in patients with acute kidney injury: a report from the national cardiovascular data registry. *Circulation.* 2012;125(3):497-504.

3. Myles PS, Smith JA, Forbes A, et al. Tranexamic Acid in Patients Undergoing Coronary-Artery Surgery. *N Engl J Med.* 2017;376(2):136-148.

4. Waldow T, Szlapka M, Haferkorn M, Burger L, Plotze K, Matschke K. Prospective clinical trial on dosage optimizing of tranexamic acid in non-emergency cardiac surgery procedures. *Clin Hemorheol Microcirc.* 2013;55(4):457-468.

5. Moussa ID, Klein LW, Shah B, et al. Consideration of a new definition of clinically relevant myocardial infarction after coronary revascularization: an expert consensus document from the Society for Cardiovascular Angiography and Interventions (SCAI). *J Am Coll Cardiol.* 2013;62(17):1563-1570.

6. Thielmann M, Sharma V, Al-Attar N, et al. ESC Joint Working Groups on Cardiovascular Surgery and the Cellular Biology of the Heart Position Paper: Perioperative myocardial injury and infarction in patients undergoing coronary artery bypass graft surgery. *Eur Heart J.* 2017;38(31):2392-2407.

7. Min JJ, Nam K, Kim TK, et al. Relationship between early postoperative C-reactive protein elevation and long-term postoperative major adverse cardiovascular and cerebral events in patients undergoing off-pump coronary artery bypass graft surgery: a retrospective study. *Br J Anaesth.* 2014;113(3):391-401.

8. Bellomo R, Kellum JA, Ronco C. Defining and classifying acute renal failure: from advocacy to consensus and validation of the RIFLE criteria. *Intensive Care Med.* 2007;33(3):409-413.

9. Shi J, Zhou C, Liu S, et al. Outcome impact of different tranexamic acid regimens in cardiac surgery with cardiopulmonary bypass (OPTIMAL): Rationale, design, and study protocol of a multicenter randomized controlled trial. *Am Heart J.* 2020;222:147-156.
